# Molecular Imprinting Technology in Quartz Crystal Microbalance (QCM) Sensors

**DOI:** 10.3390/s17030454

**Published:** 2017-02-24

**Authors:** Sibel Emir Diltemiz, Rüstem Keçili, Arzu Ersöz, Rıdvan Say

**Affiliations:** 1 Chemistry Department, Faculty of Science, Anadolu University, 26470 Eskisehir, Turkey; semir@anadolu.edu.tr (S.E.D.); arzuersoz@anadolu.edu.tr (A.E.); 2 Department of Medical Services and Techniques, Yunus Emre Vocational School of Health Services, Anadolu University, 26470 Eskisehir, Turkey; rkecili@anadolu.edu.tr; 3 Bionkit Co. Ltd., 26470 Eskisehir, Turkey

**Keywords:** molecularly imprinted polymers (MIPs), quartz crystal microbalance (QCM), biosensors, biomolecular recognition, synthetic receptors

## Abstract

Molecularly imprinted polymers (MIPs) as artificial antibodies have received considerable scientific attention in the past years in the field of (bio)sensors since they have unique features that distinguish them from natural antibodies such as robustness, multiple binding sites, low cost, facile preparation and high stability under extreme operation conditions (higher pH and temperature values, etc.). On the other hand, the Quartz Crystal Microbalance (QCM) is an analytical tool based on the measurement of small mass changes on the sensor surface. QCM sensors are practical and convenient monitoring tools because of their specificity, sensitivity, high accuracy, stability and reproducibility. QCM devices are highly suitable for converting the recognition process achieved using MIP-based memories into a sensor signal. Therefore, the combination of a QCM and MIPs as synthetic receptors enhances the sensitivity through MIP process-based multiplexed binding sites using size, 3D-shape and chemical function having molecular memories of the prepared sensor system toward the target compound to be detected. This review aims to highlight and summarize the recent progress and studies in the field of (bio)sensor systems based on QCMs combined with molecular imprinting technology.

## 1. Introduction

Biomolecular recognition plays a crucial role in biological systems where the enzyme-substrate, DNA-protein and antibody-antigen interactions are carried out [1]. These interactions and binding phenomena are usually based on lock and key models where receptors and substrates specifically interact with each other. These specific interactions include non-covalent interactions such as hydrogen bonding, metal coordination, hydrophobic interactions, Van der Waals interactions, π-π interactions and electrostatic interactions [2]. This specific molecular recognition phenomenon is commonly used in biosensor applications. For this purpose, antibodies are used as the recognition elements in biosensors since they have high selectivity and sensitivity toward the target compound. However, antibodies display some drawbacks of such as high cost and low stability under extreme conditions (higher pH, temperature and pressure).

**Figure 1:**
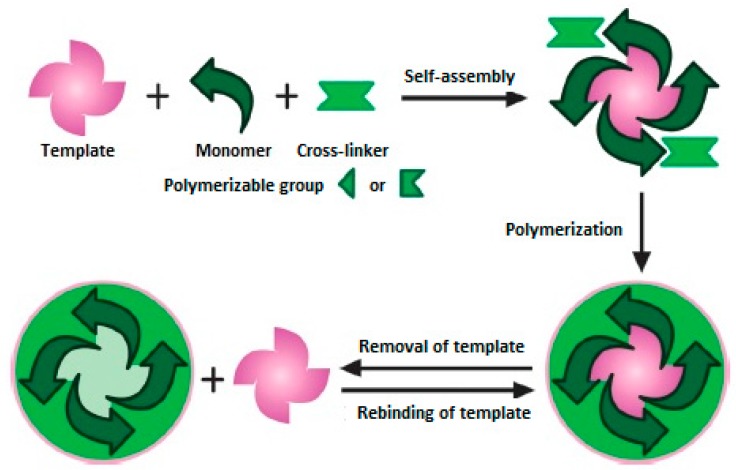
Schematic representation of molecular imprinting (reproduced with permission from [11]).

Molecularly imprinted polymers (MIPs) also called “artificial antibodies” can overcome these disadvantages of natural antibodies. MIPs are man-made artificial materials that show high affinity and selectivity toward a target compound (“template”). MIPs are prepared by polymerization of an appropriate functional monomer and a cross-linker in the presence of a template as schematically shown in Figure 1. Since MIPs are very selective toward the target compound, they are commonly used as recognition elements in the fabrication of biosensors. MIPs have the ability to bind target compounds not only by their 3-D shape, because the incorporation of specific binding groups into the selective cavities of a polymeric network enhances its affinity and selectivity toward the target analyte [3,4,5,6,7,8,9,10]. 

On the other hand, the Quartz Crystal Microbalance (QCM), is well suited as a transducer element for chemical sensors, being rapid, easy to use, highly stable and portable. Increased mass on the gold surface, associated with the binding reaction, results in a decrease of the frequency. QCM-based sensors have been used in the detection of several analytes even in very different matrix environments.

The combination of QCMs and MIPs can be carried out by two main approaches which are immobilization of pre-prepared MIP particles and in-situ polymerization. For the immobilization of MIP particles on the surface of QCM sensors, modification of the gold sensor surface with self-assembled monolayers composed of thiol-containing compounds such as 11-mercaptoundecanoic acid is performed in the first step and then MIP particles are attached to the modified sensor surface.

In in-situ polymerization, a chemical, thermal or photochemical initiator is used [12]. Photochemical initiation has some advantages such as easy control and polymerization at room temperature. In addition, MIP layers can also be formed by electropolymerization on the surface of the QCM sensor [13]. In this strategy, the thickness of the MIP layer can easily be controlled by changing the applied voltage [14]. However, there may be some difficulties such as adhesion problems during the washing step of the prepared MIP layer on the surface. Thus, some special pre-treatment processes should be applied to increase the adhesion of MIP layers on the surface of the sensor system. In this review, we provide an overview of the recent progress and applications of MIP based-QCM sensors.

## 2. MIP Based-Quartz Crystal Microbalance (QCM) Sensors

There are many reported studies on the applications of QCM sensors based on molecular imprinting technology. Some examples from the literature are briefly described in the following sections.

### 2.1. MIP-Based QCM Sensors for Biological Applications

In the last decade, one of the most promising technical applications based on the use of MIPs has been QCM. QCM sensors have been developed for the detection of various targets such as proteins (e.g., enzymes) and cells.

**Figure 2:**
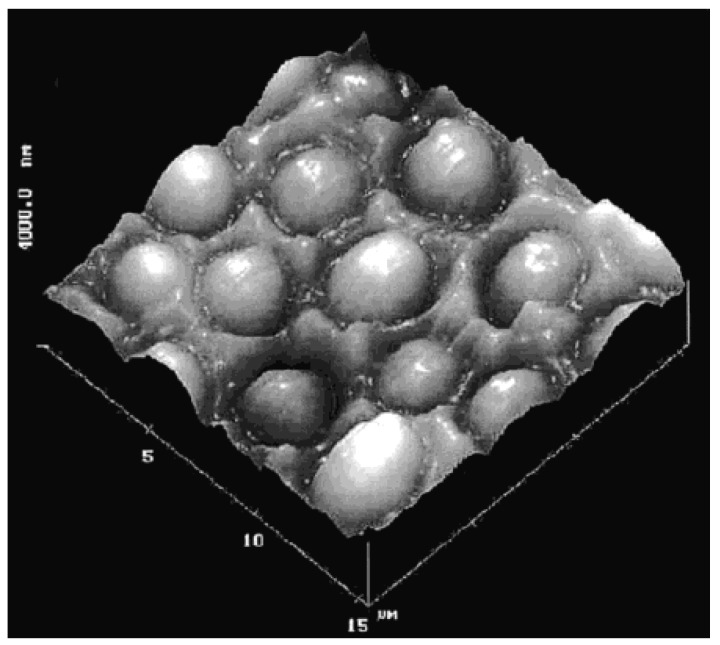
AFM image of a *Saccharomyces cerevisiae cell* imprinted sensor surface (reproduced with permission from [15]).

In one of the first reported studies performed by Dickert and Hayden [15], a MIP-based QCM sensor for selective recognition of yeast cells was prepared by surface imprinting (Figure 2). The prepared sensor exhibited recognition ability toward target yeast cells in growth media. 

QCM sensors modified with selective MIP layers also have the ability to recognize their target compounds, even in complex biological samples such as blood. Dickert and Hayden have reported another interesting study [16]. They developed a QCM sensor coated with MIP for selective recognition of erythrocytes in blood samples. The functional monomer 1-vinyl-2-pyrrolidone and template erythrocytes were polymerized in the presence of the cross-linker *N*,*N′*-methylene-bis(acrylamide) under UV-light. Then, the prepared MIP-based QCM sensor was used for the recognition of erythrocytes. The results obtained from the performance tests showed that the prepared QCM sensor has affinity and selectivity toward erythrocytes. 

Due to their size, viruses cannot be recognized using conventional optical techniques. Microbiological assays are therefore used for the detection of viruses. In this case, QCM sensors combined with MIPs prepared by surface imprinting can efficiently be used for selective detection of viruses. For example, Tai et al. have reported the first study of MIP-based QCM sensors for successful recognition of dengue virus [17]. In their study, a selective MIP for the recognition of nonstructural protein 1 was prepared on the surface of a QCM sensor. The prepared sensor was successfully applied for the recognition of nonstructural protein and the obtained results showed that the developed assay can be used for the recognition of various flaviviruses such as four different types of dengue viruses. The authors reported that additional experiments are needed to determine the diagnostic accuracy of the prepared MIP-based QCM sensor systems for the detection of acute phase dengue virus infection.

In another important study, a MIP film-coated QCM sensor for the detection of anthrax protective antigen was developed by the same researh group a few years later [18]. The results showed that the prepared MIP based-QCM sensor display high affinity toward the target epitope of anthrax protective antigen in a picomolar concentration range. This sensor platform is a fast and selective assay that can be efficiently applied for the detection of other bacterial antigens. 

**Figure 3:**
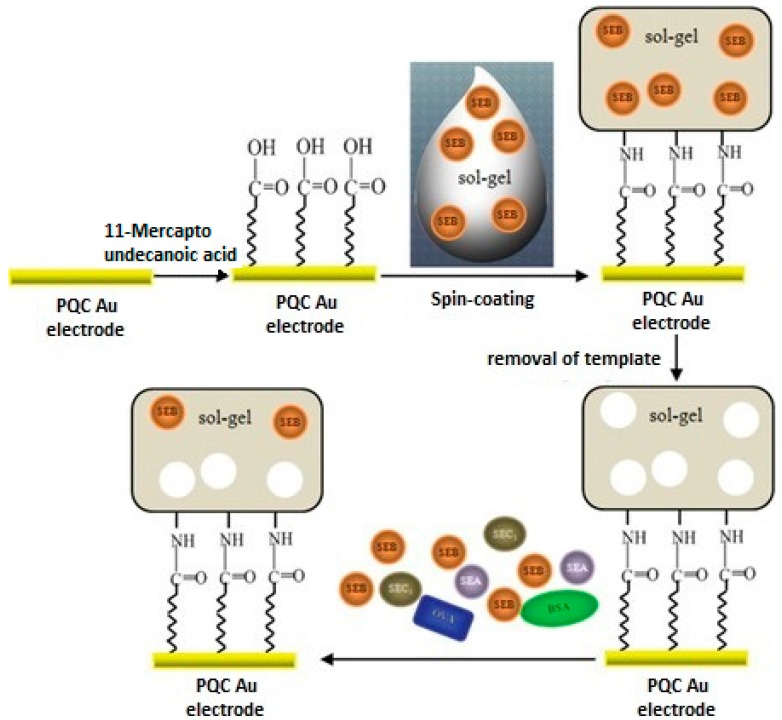
Schemes of the preparation of sol-gel imprinted thin film on the surface of the piezoelectric quartz crystal (PQC) Au-electrode for the detection of staphylococcal enterotoxin B (SEB) (reproduced with permission from [19]).

Liu et al. [19] have produced a QCM sensor for detection of staphylococcal enterotoxin B (SEB). This QCM sensor was coated by molecularly imprinted sol-gel thin film. They firstly mixed organosilanes with SEB and then this combination was coated on the sensor surface (Figure 3). Their results showed that the prepared sensor was successfully applied in the working range of 1.0 × 10^−1^ to 1.0 × 10^3^ µg·mL^−1^ and detection limit was 6.1 ng·mL^−1^. Selectivity studies were done with staphylococcal enterotoxin A (SEA), staphylococcal enterotoxin C1 (SEC1), bovine serum albumin (BSA) and ovalbumin (OVA) and the QCM sensor system exhibited high selectivity toward the templates’ analogues. These results showed that MIP-QCM combination systems are very effective tools for the determination of SEB. The studies included a comparison between the MIP-QCM combination and immunochips for the detection of SEB. Thus, they developed an alternative method to expensive immunochip systems. In the light of the obtained data the authors stated that they had developed a simple, rapid, low cost and sensitive MIP-coated QCM sensor. 

**Figure 4:**
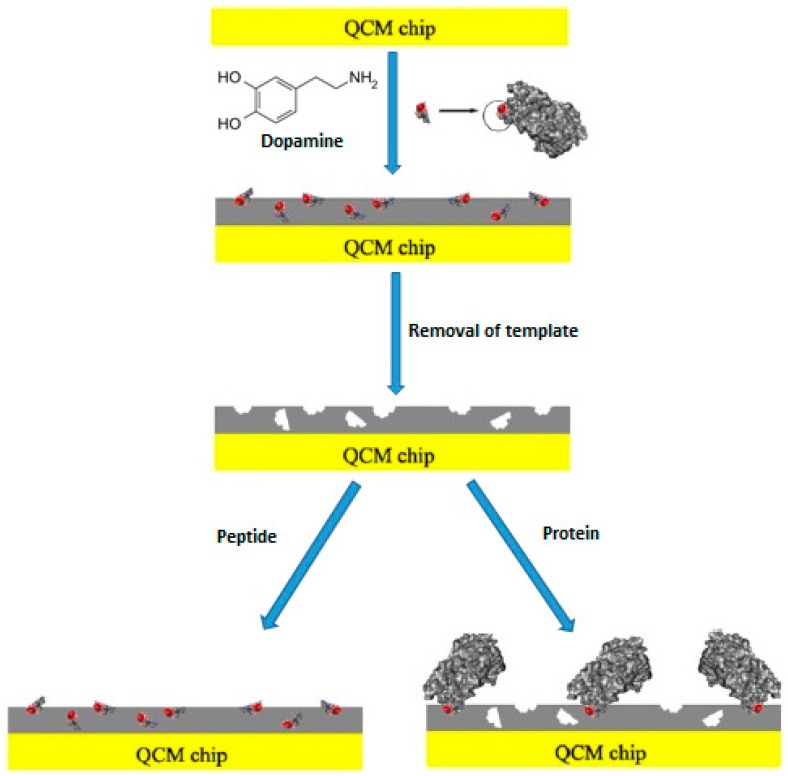
Schematic representation of epitope-imprinting (reproduced with permission from [20]).

In another study, Lu et al. [20] used a biomimetic sensor system for the determination of glycoprotein 41, gp41. Glycoprotein 41 is a protein related with human immunodeficiency virus type 1 (HIV-1). In their study the epitope-imprinting technique was used. Figure 4 shows a schematic diagram of epitope-imprinting. They also used dopamine as the functional monomer; a peptide with 35 amino acid residues of gp41 as template molecule and their report was the first study to describe the application of polydopamine in epitope-imprinting. The QCM sensor surface was coated with the hydrophilic MIP film and it was seen that this film is very selective for the template peptide and also gp41 protein. The dissociation constant (Kd) was found to be 3.17 nM, a value is very close to that obtained with monoclonal antibodies. They also investigated the analytical performance of the sensor by the imprinting effect, selectivity and real sample analysis. The presented study is very important in terms of biomolecule analysis in that it shows that small peptide groups of large biomolecule structures can be used as template molecules.

Liu et al. [21] have developed MIP-QCM-based sensors for the detection of staphylococcal enterotoxins (SEs), the cause of the most common type of food poisoning known as staphylococcal food poisoning. They used real substances for the determination of SEA and SEB. Unlike their previous study, they used QCM-D. They demonstrated that, QCM-D is more stable than QCM 200 for detection of SEA and SEB from real substances. QCM-D device is more advantageous due to the fact that the dissipation shift (ΔD) can also be measured. TEOS, APTES, OTES and template molecule- containing sol gel was coated on a QCM electrode by a spin coater. The prepared electrode was evaluated by repeated measurements of spiked milk samples. This study showed that MIP-based QCM sensors have very beneficial properties when applied in different areas such as food safety, environmental and biological applications. 

Another QCM-MIP based study was published by Phan et al. [22] in 2014. This study is very important because the authors investigated the effects of changes in cross-linker and monomer ratio on the sensitivity and response time. They have also examined the effects of different polymer preparation mechanisms such as stamp imprinting, bulk imprinting and solvent effect. For this purpose an albumin imprinted polymer was prepared on a QCM sensor. Studies have shown that a more hydrophilic polymer is obtained when acrylic acid is used instead of methacrylic acid, that when the amount of cross-linker is increased, the sensor response time is reduced and the effect of solvent change is very small. The study is very useful in terms of examining the different parameters.

In 2015, El-Sharif et al. [23] studied different acrylic amides (acrylamide, AA; *N*-hydroxymethyl-acrylamide, NHMA; *N*-isopropylacrylamide, NiPAm) and showed their effects on MIP selectivity. Their studies are composed of two parts: spectrophotometric and QCM sensor systema. The selectivity of protein-specific MIP and the control NIP was also compared towards the template molecule bovine haemoglobin (BHb). One other important result is that they achieve more selectivity and recognition capacity through the use of hydrophilic NHMA.

Boronic acids can reversibly interact with diols, α-hydroxyacids and α-amino alcohols. Due to their high binding ability toward diol-containing compounds through this feature, they are commonly used as the recognition unit of the sensor systems for the detection of carbohydrates. Immunoglobulin M (IgM) has high mannose content. Considering this, Diltemiz et al. [24] developed mannose-imprinted QCM sensors for the selective detection of mannose and IgM. The synthesis of the functional monomer methacryloylamidophenylboronic acid (MAPBA) and its use for the preparation of MIPs were performed for the first time in the literature. For the MIP film preparation on the sensor surface, a MAPBA-mannose pre-organized system was prepared. The QCM electrode surface was coated with a molecularly imprinted film by a spin-coater and the polymerization was completed under UV light. The binding affinity was evaluated by using the Langmuir isotherm and the prepared electrode has high affinity toward the analyte. This study also showed that the imprinting of mannose has the capability of determining IgM.

Latif et al. [25] used a bulk imprinting technique for the detection of the estradiols which are a kind of endocrine disrupting chemicals (EDCs). To achieve highly sensitive and selective detection even in the presence of very similar compounds that have nearly same structure, they used 17β-estradiol (E2) as a template. The interesting point of this study was the successful imprinting of bacterial cells. Therefore, both small molecules and analytes such as bacteria with their larger size could be imprinted on QCM electrode via a polyurethane layer.

l-Nicotine was also used as a template molecule in MIP-QCM studies in the literature. Alenus et al. have published two different studies on the detection of l-nicotine. One of the studies is in aqueous solution [26] while the other one is in a biological sample [27]. Although many researchers have studied l-nicotine template molecules in aqueous solution, Alenus et al. studied the detection of l-nicotine in saliva and urine. They used bulk-polymerized l-nicotine MIPs for this purpose in both studies. The monomer solutions were prepared using MAA as the functional monomer, EDMA as the cross-linker, AIBN as the initiator and l-nicotine as the template molecule, then these solutions were polymerized at 60 °C for 72 h. The obtained polymers were ground, washed to extract excess l-nicotine and then coated on a quartz crystal microbalance-dissipation (QCM-D) electrode with PVC. They demonstrated that MIPs bind 4.03 times more l-nicotine than NIPs in water and 1.99 times more in PBS at pH 9. l-Nicotine-spiked saliva and urine samples were diluted in water and PBS solution. l-Nicotine was successfully determined in samples of patients’ saliva after using nicotine gums and smokeless tobacco. This study shows that even though the l-nicotine concentration is in the micromolar range, it could be detected directly in saliva and urine samples.

**Figure 5:**
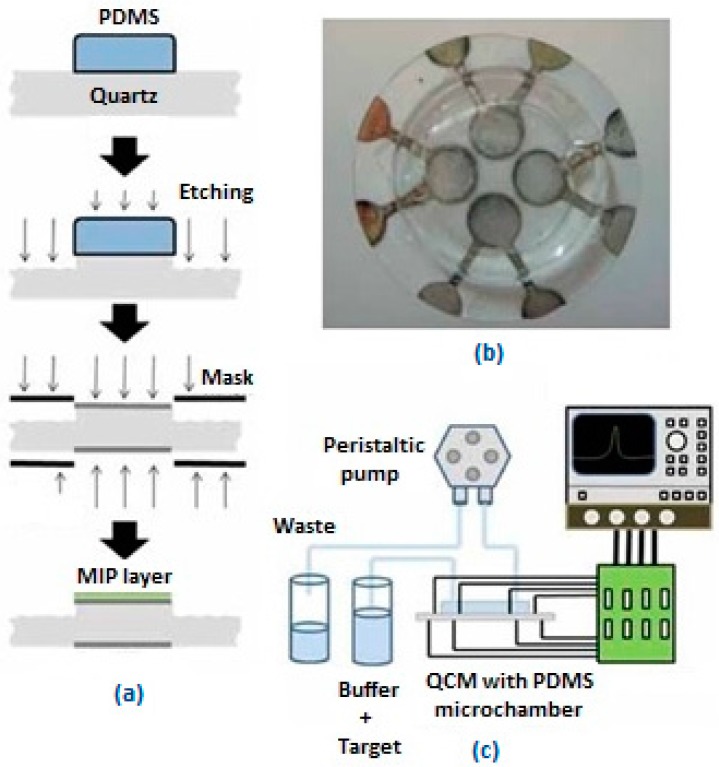
(**a**) Schematic demonstration of MIP-based QCM sensor; (**b**) Prepared MIP based-QCM sensor with multi-channel; (**c**) Schematic depiction of the prepared sensor system combined with impedance analyzer (reproduced with permission from [28]).

Another study for the determination of l-nicotine was accomplished by Croux et al. [28]. They used a different approach and application with a MIP-based QCM sensor and prepared a multi-channel system. Their four channel system contains two NIP/MIP pairs. The mixture of MAA, EGDM, AIBN and template molecule l-nicotine was placed on the QCM electrode via a PDMS mold (Figure 5). Finite element analysis (FEA) was used for this study to investigate the flow inside the system. In this way turbulence causing concentration problems was prevented.

Electropolymerization is another crucial approach for the preparation of MIPs on the surface of QCM sensors. In situ polymerization strategies can be used for this purpose. The first attempts using phenolic functional monomers were carried out to investigate the effect of electropolymerization on MIP film preparation, but the obtained MIP film layers were thick and non-specific interactions had to be blocked using other compounds [29,30]. Considerable progress has been achieved in this area by depositing thin film layers at a certain point of the sensor surface [31]. One of the main advantages of the electropolymerization technique is that the thickness of the MIP film layer can be changed by applying different voltage values. Lenain and co-workers [32] first prepared an electrode composed of molecularly imprinted sub-micron spherical particles for the recognition of metergoline. Hybrid structures can also be prepared by using MIPs with proteins or a self-assembled monolayer. The first combination of an enzyme with a MIP film on the sensor surface for the detection of electroactive proteins was reported by the research group of Yarman [33]. 

Apodaca et al. [34] have developed a MIP-based QCM sensor for the selective recognition of folic acid. MIP film using bisterthiophene dendrons and folic acid as the template was prepared by electropolymerization on the sensor surface. The analytical performance of the prepared sensor was investigated in the presence of pteroic acid, caffeine and theophylline. The obtained results showed that the prepared MIP-based QCM sensor exhibited high affinity and selectivity toward target compound folic acid. The detection limit was found to be as 15.4 µM.

It is also possible to detect target proteins in biological samples by using MIP-based QCM sensors. If the template is a large biomolecule such as an enzyme or protein, the surface imprinting strategy is used. In another remarkable study, a MIP-based QCM sensor selective to ribonuclease A was developed by Liu and his research group [35]. 

**Figure 6:**
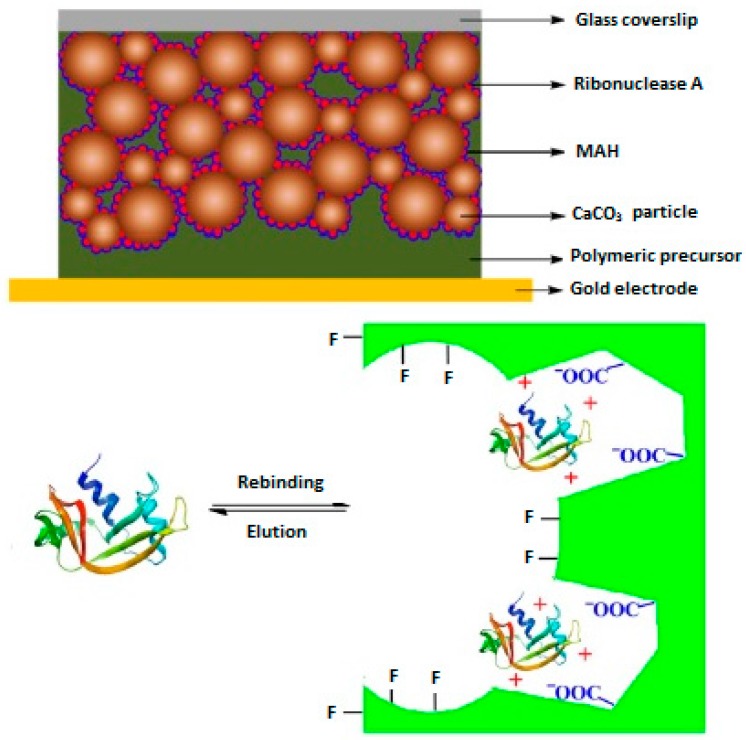
Preparation of MIP film coated QCM sensor for Ribonuclease A (Reproduced with permission from Reference [35]).

In their previous attempts, they prepared calcium carbonate nanoparticles as the porogen for the preparation of MIP thin layers and the obtained results showed that the synthesized polymeric film exhibited high porous effects. Considering this, a MIP film responsive to ribonuclease A was prepared on a gold sensor surface using a surface imprinting approach by polymerization of ribonuclease A template and CaCO_3_ nanoparticles in the presence of methacryloylamidohistidine (MAH) and trimethylolpropane trimethacrylate (TRIM) as the functional monomer and cross-linker, respectively. Figure 6 shows the schematic representation of the prepared QCM sensor coated with ribonuclease A MIP film. The results obtained from the experiments on the performance of the prepared QCM sensor toward ribonuclease A showed that the prepared sensor exhibits high selectivity toward the target protein ribonuclease A in the presence of lysozyme as the control protein. The selectivity factor was calculated to be 3.3 at the protein concentration of 1−10^−6^ g·mL^−1^.

**Figure 7:**
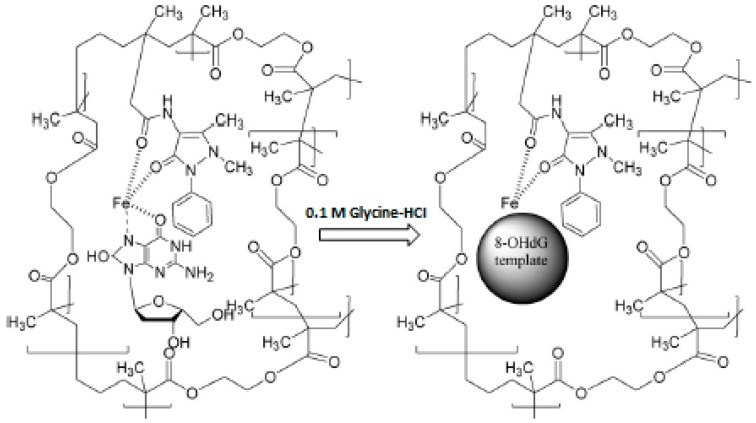
Preparation of MIP for 8-OHdG (reproduced with permission from [36]).

The chelating properties of various metal ions with the desired compounds are also used for the preparation of the selective MIPs. Considering this feature, MIPs prepared by using metal-chelate functional monomers were designed for the construction of MIP-based QCM sensors toward biological compounds in real samples. For example, Ersöz et al. [36] have prepared a QCM sensor composed of 8-hydroxy-2’-deoxyguanosine (8-OHdG) imprinted film for the detection of 8-OHdG levels in biological samples. For this purpose, MIP film selective to 8-OHdG was synthesized by using a photo-graft surface polymerization technique. Methacryloylamidoantipyrine-iron, *N*-*N*′-methylenebisacrylamide and 8-OHdG were used as the complex functional monomer, cross-linker and template, respectively. The preparation of a MIP-based QCM sensor toward 8-OHdG is schematically represented in Figure 7. The results obtained from the performance studies of the prepared sensor showed that the prepared MIP-based QCM sensor displays high affinity and selectivity toward 8-OHdG. The affinity constant (K_a_) of the sensor was found to be 48,510 M^−1^.

In a study reported by Lee et al. [37] MIP film-coated QCM sensors were used for the selective recognition of lipase, amylase and lysozyme which are digestive enzymes found in saliva. For this purpose, the sensor surface was coated with a mixture of target protein and poly(ethylene-co-vinyl alcohol). Then, a MIP thin film was prepared applying the thermally induced phase separation approach. The prepared sensors were applied to recognize lipase, amylase and lysozyme in real samples. The obtained results showed that the prepared MIP-based QCM sensors have the ability to recognize target proteins at lower concentrations and the limit of detection values were found to be 7 pM, 2.5 pM and 3.5 pM for lysozyme, lipase and amylase, respectively.

**Figure 8:**
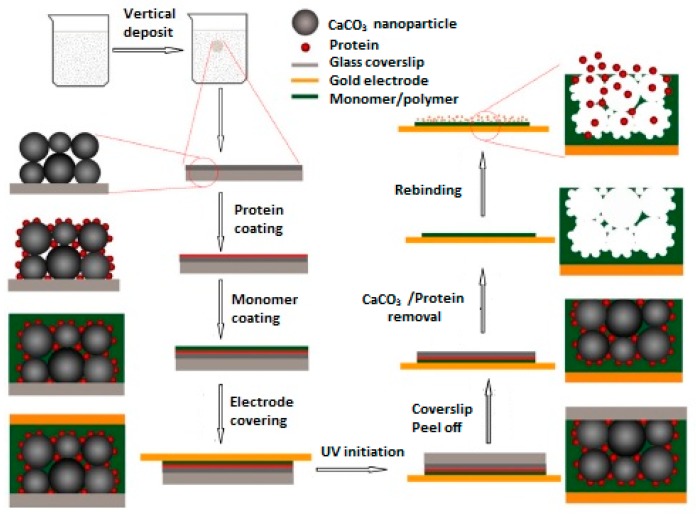
Preparation of QCM sensor toward lysozyme (reproduced with permission from [38]).

A QCM sensor coated with lysozyme imprinted macroporous film was developed by Zhou and co-workers [38]. In their study, methyl methacrylate (MMA) and TRIM were used as the functional monomer and cross-linker, respectively. In addition, CaCO_3_ nanoparticles were used as porogen to form interconnected macropores in the MIP film on the QCM sensor surface (Figure 8). The results of experiments for the selective recognition of target protein lysozyme by using the prepared MIP-based QCM sensor showed that the prepared sensor displays high affinity and selectivity toward lysozyme in the presence of myoglobin. The selectivity factors were calculated as 3.1, 4.0 and 3.6 at protein concentrations of 5 × 10^−5^ g·mL^−1^, 20 × 10^−5^ g·mL^−1^ and 50 × 10^−5^ g·mL^−1^, respectively.

Mirmohseni et al. [39] have reported the preparation of a MIP-based QCM sensor for the selective detection of phenylalanine. MIP film sensitive toward phenylalanine on the surface of the sensor was synthesized by co-polymerization of acrylonitrile (AN) and acrylic acid (AA) [poly(AN-co-AA)] in the presence of the target amino acid phenylalanine. The prepared MIP-based QCM sensor exhibited a linear response toward phenylalanine in the concentration range of 50–500 mg·L^−1^. The limit of detection was found to be 45 mg·L^−1^. 

In another study, a thymine-imprinted thin film-coated QCM sensor was developed by Diltemiz et al. [40]. For this purpose, methacryloylamidoadenine (MA-Ade) and thymine were used as the functional monomer and template compound, respectively. Polymerization was carried out under UV-light to form allyl-based self-assembled monolayers (SAMs) which have rougher surfaces compared to traditional monolayers formed by thiol modification. This approach provides an efficient recognition of DNA. The prepared MIP-based QCM sensor was used for the detection of thymine in the presence of uracil nucleobase. The Langmuir binding isotherm was applied to investigate the binding behavior of the prepared QCM sensor. The obtained results showed that the prepared sensor has homogeneous binding sites and displays high affinity toward the target compound thymine and K_a_ value was calculated as 1.0 × 10^5^ M^−1^. This reported approach offers a cost-effective, easy and sensitive MIP-based sensor system based on mimicking of DNA. The prepared allyl-based SAMs are great choice for the optimization of QCM sensors coated with MIP layers which exhibit high potential to become a commercial products. 

In 2015, Jha and Hayashi have prepared a MIP coated-QCM sensor for the recognition of aldehyde compounds in body odor [41]. Gas chromatography-mass spectrometer (GC-MS) was applied for the characterization of odor samples. The functional monomer polyacryclic acid (PAA) with presence of a template aldehyde was polymerized on the sensor surface. The obtained results showed that the prepared MIP-based QCM sensor for heptanal exhibits high sensitivity, fast response and reproducibility compared to other prepared sensors toward hexanal and nonanal.

### 2.2. MIP-Based QCM Sensors for Food and Beverage Applications

Food and beverage industries need sensitive and reliable analytical techniques for the quality control of their products. During the production process, continuous monitoring should be carried out for the food safety. Traditional approaches such as enzyme assays and immuno techniques based on natural antibodies are commonly used for the analysis of food samples, but these techniques have some drawbacks such as high cost, low stability. QCM sensors combined with MIPs as plastic antibodies can be an alternative approach and overcome these drawbacks of traditional approaches. 

For example, Sun et al. [42] have reported the preparation of MIP film-coated QCM sensors for the selective recognition of quinine and saccharine in bitter drinks. The functional monomer MAA and cross-linker (EDMA) were polymerized in the presence of template compounds quinine and saccharine to obtain selective MIP films on the sensor surface. Then, recognition performance of the prepared sensors toward target compounds was investigated. Other possible interfering compounds such as vanillin, caffeine, citric acid, sodium benzoate, sodium bicarbonate and sucrose were used to test selectivity of the prepared MIP-based QCM sensor. The obtained results from the experiments showed that the prepared sensor systems have high sensitivity (2.04 mg·L^−1^ and 32.8 mg·L^−1^ for quinine and saccharine, respectively). The MIP-coated QCM sensor systems were developed to provide a sensitive, facile and fast method for the determination of quinine and saccharine in tonic water at a practical concentration range with fast sample throughput and sufficient repeatability. MIP film-coated QCM technology thus provides a promising methodology for the taste application in flavor forecast and quality control of experimental, intermediate and final products for food, drinks and beverages.

In another important study, Iqbal et al. [43] have developed QCM sensors with polystyrene- based MIP membranes for the determination of different terpenes (limonene, α-pinene, β-pinene, estragole, eucalyptol and terpinene) in fresh and dried herb samples. The prepared sensor systems were succesfully applied to recognize target terpenes in rosemary, basil and sage. The sensitivity of the prepared sensors was <20 ppm of target compounds and the linear concentration range of the sensor response was <20 ppm to 250 ppm. Are the following really food and beverage applications?

The research group of Dickert [44] has developed a QCM sensor coated with MIP film for selective detection of tobacco mosaic virus in tobacco plant sap. MIP film on the sensor surface was prepared by polymerization of the functional monomer MAA, cross-linker EDMA and template virus. The prepared sensor was successfully used for the recognition of the target virus in real samples. The results showed that the prepared MIP-based QCM sensor exhibits high recognition ability toward tobacco mosaic virus in the concentration range of 10 ng·mL^−1^ to 1.0 mg·mL^−1^ and that qualitative detection of virus in infected leaf sap was successfully achieved. In this study, the authors also showed that the prepared sensor based on biomimetic polymers in combination with QCM is a new approach for monitoring of plant viruses directly in the plant sap within minutes. Ebarvia et al. [45] has prepared a MIP-based QCM sensor for the selective detection of chloramphenicol. In their work, selective MIP toward chloramphenicol was prepared by using precipitation polymerization and MAA, TRIM and chloramphenicol were used as the functional monomer, cross-linker and the template, respectively. MIP suspension in polyvinylchloride-tetrahydrofuran solution was coated onto the 10 MHz AT-cut quartz crystal by spin-coating. The obtained results showed that the prepared MIP based QCM sensor exhibits high affinity toward chloramphenicol.

In another study published in 2015 by Eren et al. [46], a MIP based-QCM sensor for selective determination of lovastatin (LOV) in red yeast rice was developed by formation of allylmercaptane monolayer on the sensor surface. For this purpose, LOV imprinted poly(2-hydroxyethyl methacrylate-methacryloylamidoaspartic acid) [p(HEMA-MAAsp)] nanofilm was prepared on the surface of a QCM sensor. The obtained results from the performance tests of the prepared sensor showed that the linearity range was 0.10–1.25 nM and the limit of detection was found as 0.030 nM. 

In 2014, Dai et al. [47] developed a novel material based on MIP-QCM sensor for histamine in food samples. Histamine (HA) is a critical marker of food quality, being an indicator of bacterial contamination. The obtained results showed that the sensor exhibited linear behavior for HA concentrations of 0.11 × 10^−2^ to 4.45 × 10^−2^ mg·L^−1^, a detection limit of 7.49 × 10^−4^ mg·kg^−1^ (S/N = 3), and high selectivity for HA (selectivity coefficient > 4) compared with structural analogues, good reproducibility, and long-term stability were observed. Also, the sensor was used to determine the concentration of HA in spiked fish products and the recovery values were found be 93.2%–100.4%. These outstanding detection limits are highly suitable for on-line monitoring of small amounts of histamine.

In another study published by Yan et al. [48], a QCM sensor was coated with MIP film for the determination of daminozide, which is a potential carcinogen in apple samples. For the daminozide sensor preparation, a MIP film using MAA as the functional monomer and EDMA as the cross-linker was prepared in the presence of the target molecule. The obtained results from the performance studies of the prepared MIP based-QCM sensor showed that the sensor has high selectivity and affinity toward the target compound daminozide in real samples. The limit of detection was found to be 5.0 × 10^−8^ mg·mL^−1^.

Sun and Fung [49] prepared MIP based-QCM sensor systems for pirimicarb residues in vegetables which are potentially mutagenic and carcinogenic. For this purpose, they synthesized three different MIP particles using the functional monomer MAA by bulk and precipitation polymerization techniques. Then, the prepared MIP particles were coated on the surface of the QCM sensor. The obtained results showed that the nanoscale MIP particles prepared by precipitation polymerization were the best coating material for the sensor surface. The recognition performance of the prepared MIP based-QCM sensor toward pirimicarb in the presence of other interfering compounds such as atrazine, carbaryl, carbofuran and aldicarb was investigated. The prepared sensor showed high selectivity toward pirimicarb in the linear working range of 5.0 × 10^−6^ to 4.7 × 10^−3^ M and the limit of detection was calculated as 5.0 × 10^−7^ M. 

Avila et al. [50] developed a QCM sensor coated with MIP for the detection of vanillin in vanilla sugar samples. Other similar compounds such as 4-hydroxybenzyl alcohol, 4-hydroxy-3-methoxy-benzyl alcohol and 4-hydroxybenzaldehyde as potential interferences were used for the selectivity experiments. The obtained results showed that the prepared sensor has high affinity and selectivity toward vanillin. The linear concentration range for the sensor response was 5 to 65 µM.

In another study, a MIP based-QCM sensor for citrinin was prepared by Fang and co-workers [51]. They developed a novel 3D-composite QCM sensor composed of MIP/Au nanoparticles/mesoporous carbon. The prepared sensor was successfully applied for the detection of citrinin in several cereals such as rice, white rice vinegar and wheat. The limit of detection was found as 1.8 × 10^−9^ M. 

### 2.3. MIP-Based QCM Sensors for Environmental Applications

Environmental pollution is one of the crucial and challenging problem in the world today. Many compounds found in water and soil such as heavy metals, phenolics, pesticides, herbicides and pharmaceuticals, etc. are hazardous for humans, plants, animals and may cause serious health problems. Therefore, it is important to detect and remove these compounds from environmental samples. Conventional techniques such as HPLC, GC and CE have been applied for the analysis of these compounds in environmental samples [52,53,54,55]. However, these approaches are quite expensive, time consuming and require experienced researchers. These disadvantages of conventional approaches can be overcome by design and contruction of MIP-based QCM sensor systems that have high affinity and selectivity toward desired analyte in complex matrices.

One of the first examples of a MIP-based QCM sensor for environmental applications was reported by Percival et al. [56]. In their study, a selective QCM sensor coated with MIP thin film sensitive toward l-menthol in aqueous solutions was prepared. To obtain a selective MIP for l-menthol, MAA, l-menthol and EDMA were used as the functional monomer, template and cross-linker, respectively. The prepared thin MIP film was coated on the surface of QCM by using a sandwich casting approach. The limit of detection was found to be 200 ppb within a response range of 0 to 1.0 ppm. 

An interesting work regarding the use of MIPs in QCM sensors was performed by Iglesias and his colleagues in 2009 [57]. They prepared a hybrid system composed of a polyurethane based-MIP coated QCM sensor for selective recognition and separation in chromatographic systems. The prepared hybrid system was successfully applied for selective recognition and separation of benzene, toluene, ethylbenzene, and xylenes in gasoline vapors. 

In another interesting study reported by Dickert et al. [58], a QCM sensor coated with a polyurethane based-MIP film was developed for detection of degradation products in automotive engine oils. 

These reported examples show that QCM sensors combined with MIPs selective to target compound/s can be succesfully used for the desired analyte/s in complex matrices in liquid and gas phases. 

**Figure 9:**
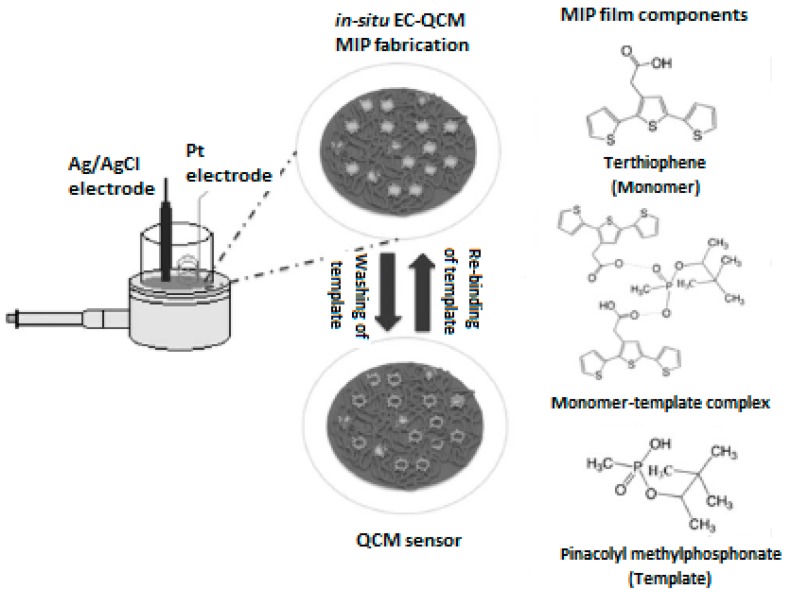
MIP-based QCM sensor toward pinacolyl methylphosphonate (reproduced with permission from [59]).

The recognition of chemical nerve agents has also been successfully achieved by using MIP-based QCM sensors. In 2012 Vergara et al. [59] developed an electrochemically MIP polythiophene film QCM surface for selective and sensitive detection of pinacolyl methylphosphonate (PMP). The lectrochemistry-QCM (EC-QCM) technique was used for the deposition of the MIP film onto the electrode. Figure 9 shows the schematic depiction of the MIP-based QCM sensor toward PMP. The synthesis was performed using cyclic voltammetry (CV) techniques applying apotential in the range of 0 to 1100 mV. 

Organophosphate pesticides are able to cause neurological diseases and are considered toxic compounds. Paraoxon is the most commonly used organophosphate pesticide. Novel MIPs-based QCM biosensors for paraoxon recognition have been investigated by Özkütük et al. [60]. In their study, chitosan-Cd(II) (TCM-Cd(II)) modified with thiourea and epichlorohydrin were used as the functional monomer and cross-linker, respectively. The obtained results showed that the prepared QCM sensor-coated MIP film exhibited high affinity toward paraoxon in the concentration range of 0.02 to 1 μM and the limit of detection was found to be 0.02 μM. In another work [61], they used N-(2-aminoethyl)-3-aminopropyltrimethoxysilane–Cu(II) (AAPTS–Cu(II)) as a new metal–chelating monomer and tetraethoxysilane (TEOS) crosslinking agent for the polymerization.

The MIP-based QCM biosensor technique was applied to the detection of some drugs by Eslami et al. [62] and Kim et al. [63] in 2015. The nanostructured conducting MIP film was synthesized by CV method on the QCM electrode. The electrode was successfully applied for selective detection of ibuprofen in sample solutions. 

**Figure 10:**
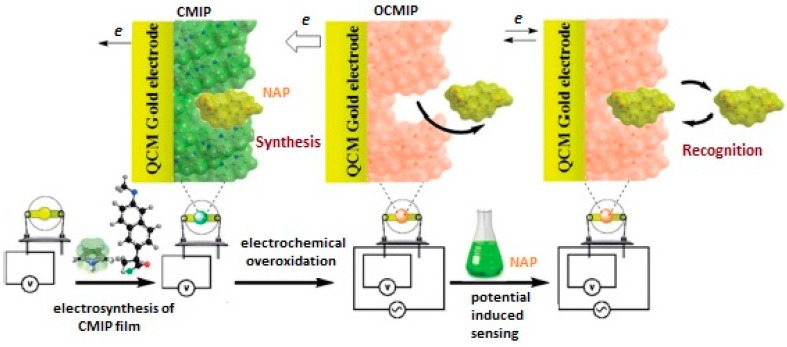
MIP-based QCM sensor for naproxen (reproduced with permission from [64]).

In another study, a QCM sensor was coated with nano-MIP film for the determination of naproxen (NAP) [64]. MIP film preparation was performed on the surface of gold electrode by polymerization of pyrrole in the presence of template compound NAP as schematically shown in Figure 10. Scanning electron microscopy (SEM), infrared spectroscopy (FT-IR), cyclic voltammetry (CV) and electrochemical impedance spectroscopy (EIS) were used for the characterization of the prepared sensor. The obtained results from performance tests of the MIP-based QCM sensor toward NAP showed that the prepared sensor shows high affinity and selectivity toward NAP. The limit of detection was calculated to be 0.04 µM. 

Gao et al. developed QCM sensors coated with ultra-thin MIP films for the detection in tap water of profenofos, which is an organophosphorus pesticide [65]. The sensor systems were prepared by using entrapment and in-situ self-assembly approaches. The results showed that the best recognition performance was obtained by a MIP-based QCM sensor prepared by in-situ self-assembly. Selectivity studies were also carried out in the presence of other potentially interfering compounds such as chlorpyrifos, parathion dichlorvos and omethoate. The prepared MIP based-QCM sensor displayed high affinity and selectivity toward profenofos in real samples. The limit of detection was calculated as 2.0 × 10^−7^ mg·mL^−1^.

Another important study was performed by He and co-workers [66]. In their study, MIP-based QCM sensors were prepared for selective recognition of microcystin-LR (MC-LR) (a toxic peptide produced by some types of algae) in lake water. For this purpose, MAA, MC-LR and EDMA were used as the functional monomer, template and cross-linker, respectively. Selectivity of the prepared MIP film coated sensor was investigated in the presence of MC-RR, MC-RY and nodularin as interfering compounds. The obtained results showed that the prepared sensor has high selectivity toward target MC-LR in the presence of interfering compounds in lake water. The limit of detection was found as 0.04 nM. In this study, the investigation of binding performance of the prepared sensor toward target in real samples shows the potential practical use of the prepared sensor. 

In another study, a MIP coated-QCM sensor was prepared for the detection of atrazine in wastewater samples [67]. For the preparation of MIP film on the sensor surface, HEMA, atrazine and EDMA were used as functional monomer, template and cross-linker respectively. The prepared MIP based-QCM sensor showed high affinity and sensitivity toward target compound atrazine. The obtained linear concentration range was 0.08 to 1.5 nM and the limit of detection was calculated as 0.028 nM. Recently reported studies of MIP-based QCM sensors in different applications are given in Table 1.

## 3. Conclusions and Future Trends

The examples described in this review highlight the recent progress and applications of MIP based-QCM sensors over the past years. The growing number of reported studies in which QCM sensor systems based on the molecular imprinting technique have been used in various application areas showed that these sensor systems are promising for selective recognition. QCM sensors with high specificity and sensitivity are commonly used as monitoring tools for target compounds in complex matrices where the selectivity is crucial. The combination of QCM sensors with target molecule memories having MIP thin films through the pre-recognition provides affinity toward the target compound, highly selective binding sites and novel, more sensitive sensing systems based on homogeneity in a larger number of recognition sites in MIPs. This combination has led to the design and development of a next generation of sensor platforms providing useful information for the progress of analytical sciences. On the other hand, MIP-coated QCM sensor platforms can also be potentially applied to process control and monitoring, assistance in the development of new products, as well as to the assessment of synergistic effects of food, drug, artificial enzyme and inhibitors and other innovative products.

## Figures and Tables

**Table 1:** Recent reported studies of MIP-based QCM sensor in different applications.

Reference	Composition of QCM Sensor	Target	Sample
**Applications to Environmental Samples**			
[68]	MIP film prepared by using functional monomer MAA on the sensor surface	Propranolol	Aqueous solutions
[69]	MIP film prepared by using functional monomer MAA on the sensor surface	Cu^2+^ and Ni^2+^ ions	Aqueous solutions
[70]	MIP film prepared by using functional monomer MAA on the sensor surface	Cu^2+^	Wastewater
[71]	MIP film prepared by using functional monomer pyrrole on the sensor surface	Trichloroacetic acid	Drinking water
[72]	MIP film prepared by using functional monomer MAA on the sensor surface	Methomyl	Natural water
[73]	MIP film prepared by using functional monomer 3-thiophene acetic acid (3-TAA) on the sensor surface	Melphalan	Aqueous solutions
[74]	Cyclodextrin-modified poly(L-lysine) based- MIP film on the sensor surface	Bisphenol A	Aqueous solutions
**Applications to Biological Samples**			
[75]	MIP film prepared by using functional monomer 1-Vinyl-2-pyrrolidone on the sensor surface	Heparin	Human plasma
[76]	MIP film prepared by using functional monomer MAA and poly(amidoamine) dendrimer on the sensor surface	Methimazole	Human urine
[77]	MIP film prepared by using functional monomer methacryloylamido tryptophan on the sensor surface	Bilirubin	Human plasma and Urine
[78]	MIP film prepared by using functional monomer methacryloylamido histidine on the sensor surface	Cholic acid	Human serum and Urine
[79]	MIP film prepared by using 3-dimethylaminopropyl methacrylamide as the functional monomer on the sensor surface	Albumin	Human serum
[80]	MIP film prepared by using functional monomer MAA on the sensor surface	D-Methamphetamine	Human urine
**Applications to Food and Beverage Samples**			
[81]	MIP microsphere modified QCM sensor	Endosulfan	Drinking water and milk
[82]	MIP/poly(*o*-aminothiophenol) membrane/Au nanoparticles composite on the sensor surface	Ractopamine	Swine feed
[83]	MIP film prepared by using functional monomer methacryloylamido antipyrine on the sensor surface	Caffeic acid	Tea, apple and potato
[84]	MIP film on the surface of the alkanethiol modified-gold electrode	Thiacloprid	Celery Juice
[85]	Gold electrode coated with molecularly imprinted nanoparticles prepared by using functional monomer methacryloylamido histidine	Lysozyme	Chicken egg white
[86]	MIP film prepared by using methacryloylamidoaspartic acid as the monomer on the sensor surface	Kaempferol	Orange and apple juice
